# The p53-Target Gene *Puma* Drives Neutrophil-Mediated Protection against Lethal Bacterial Sepsis

**DOI:** 10.1371/journal.ppat.1001240

**Published:** 2010-12-23

**Authors:** Sean P. Garrison, Justin A. Thornton, Hans Häcker, Richard Webby, Jerold E. Rehg, Evan Parganas, Gerard P. Zambetti, Elaine I. Tuomanen

**Affiliations:** 1 Department of Biochemistry, St. Jude Children's Research Hospital, Memphis, Tennessee, United States of America; 2 Department of Infectious Diseases, St. Jude Children's Research Hospital, Memphis, Tennessee, United States of America; 3 Department of Pathology, St. Jude Children's Research Hospital, Memphis, Tennessee, United States of America; Tufts University School of Medicine, United States of America

## Abstract

Disruption of p53/Puma-mediated apoptosis protects against lethality due to DNA damage. Here we demonstrate the unexpected requirement of the pro-apoptotic p53-target gene *Puma* to mount a successful innate immune response to bacterial sepsis. *Puma^−/−^* mice rapidly died when challenged with bacteria. While the immune response in *Puma^−/−^* mice was unchanged in cell migration, phagocytosis and bacterial killing, sites of infection accumulated large abscesses and sepsis was progressive. Blocking p53/Puma-induced apoptosis during infection caused resistance to ROS-induced cell death in the CD49d+ neutrophil subpopulation, resulting in insufficient immune resolution. This study identifies a biological role for p53/Puma apoptosis in optimizing neutrophil lifespan so as to ensure the proper clearance of bacteria and exposes a counter-balance between the innate immune response to infection and survival from DNA damage.

## Author Summary

Protection against fulminant, lethal bacterial sepsis requires a robust and rapid response from the neutrophil subclass of white blood cells. However, prolonged survival of these activated cells leads to organ damage. The cellular processes determining the optimal life span of neutrophils are not understood. The p53/Puma pathway drives programmed cell death by initiating apoptosis. This response is beneficial in preventing cellular transformation by eliminating abnormal cells that have sustained DNA damage. Here we demonstrate an opposing effect of the pathway in the context of a successful immune response to bacterial sepsis. *Puma^−/−^* mice rapidly died when challenged with bacteria and sites of infection accumulated large abscesses. Blocking p53/Puma-induced apoptosis during infection prolonged the life of the CD49d+ neutrophil subpopulation, resulting in insufficient immune resolution. This study expands the biological role of p53/Puma-induced apoptosis to the proper clearance of bacteria by neutrophils and exposes a counter-balance between the innate immune response to infection and survival from DNA damage.

## Introduction

Programmed cell death is critical to the course of natural development and for the elimination of potentially abnormal cells that have encountered various stresses such as DNA damage, oncogene overexpression or hypoxia [1]. During cellular stress, the p53 tumor suppressor protein blocks cell cycle progression allowing time to repair the damage [2], [3] or induces apoptosis largely through the upregulation of the Bcl-2 family BH3-only protein Puma (p53 upregulated modulator of apoptosis) [4]–[6]. In the absence of p53/Puma-induced cell death, animals and primary cell cultures demonstrate remarkable resistance to apoptosis following DNA damage, glucocorticoid treatment, and cytokine withdrawal [7]–[9]. Despite this survival advantage, *Puma* is maintained in the genome suggesting the existence of a strongly positive, counteracting selective pressure. We hypothesized that one such ubiquitous selective pressure might arise in the context of infection. During infection, apoptosis has generally been regarded as a consequence of cellular injury. For instance, bacterial-induced apoptosis is a major determinant of cellular and organ injury during infections of the brain and gut [10], [11] and inhibition of apoptosis improves residual organ function in survivors [12], [13]. Clinical trials have indicated that sequelae of bacterial meningitis are mitigated by pharmacological inhibition of bacterial-induced neuronal apoptosis [14]. Conversely, widespread apoptosis of phagocytic cells, as seen with chemotherapy, impairs control of an infection [15], [16]. These results would suggest that loss of *p53* or *Puma* would potentially benefit outcome of infection by preserving host cells and organ function. However, there are some suggestions that blocking apoptosis is not universally beneficial in the context of infection. Inhibition of iNOS was associated with decreased macrophage apoptosis but increased mortality in bacterial pneumonia [17]. In septic mice, absence of p53 decreased apoptosis but accelerated mortality in one study [18] while no effect on outcome was seen in another [19]. Active interruption of apoptosis by Epstein Barr virus or *Chlamydia* prolongs host cell survival but enhances pathogenesis [20], [21]. This dichotomy of beneficial vs. harmful effects of disrupting apoptosis is reflected in the innate immune response to infection where leukocytes clear bacteria, but then apoptosis of phagocytes promotes immune resolution and wound repair [22], [23]. Therefore the timing of survival factor expression in phagocytes would be expected to be critical to both successful elimination of the pathogen and resolution of inflammation.

Spontaneous apoptosis is known to be a major determinant of the short neutrophil life span [24], [25]. Neutrophils in culture die rapidly, but when incubated with interleukins survive longer (t ½ 35 vs. 115 h). Early in infection, neutrophil survival is promoted through the upregulation of Mcl-1 (Bcl-2 pro-survival member), which allows cells to survive longer in septic patients [26]. Increased survival also occurs if neutrophils are incubated with specific bacterial components, an effect mediated through apoptotic prevention [27]. While extended survival of phagocytes ensures an adequate number of cells to respond to infection, once phagocytosis occurs, pro-apoptotic genes are upregulated and anti-apoptotic genes are downregulated with the resultant induction of apoptosis. This process promotes resolution of inflammation through neutrophil clearance by resident macrophages and increased expression of the anti-inflammatory cytokines TGF-β and interleukin-10 [15], [22]. Despite decades of investigation, many of the mechanisms controlling the cell fate decisions of these important innate cells remain elusive. We sought to map the role of pro-apoptotic factors in specific functions of neutrophils during the course of infection using a model of acute pneumonia and sepsis caused by *Streptococcus pneumoniae*, a gram-positive invasive bacterium. Here we report that, despite dramatic protection against DNA damage, the loss of either Puma or p53 is associated with a shift to overwhelming lethal sepsis in the context of bacterial infectious diseases.

## Results

### Loss of *Puma* compromises control of pneumococcal infection

**Figure 1:**
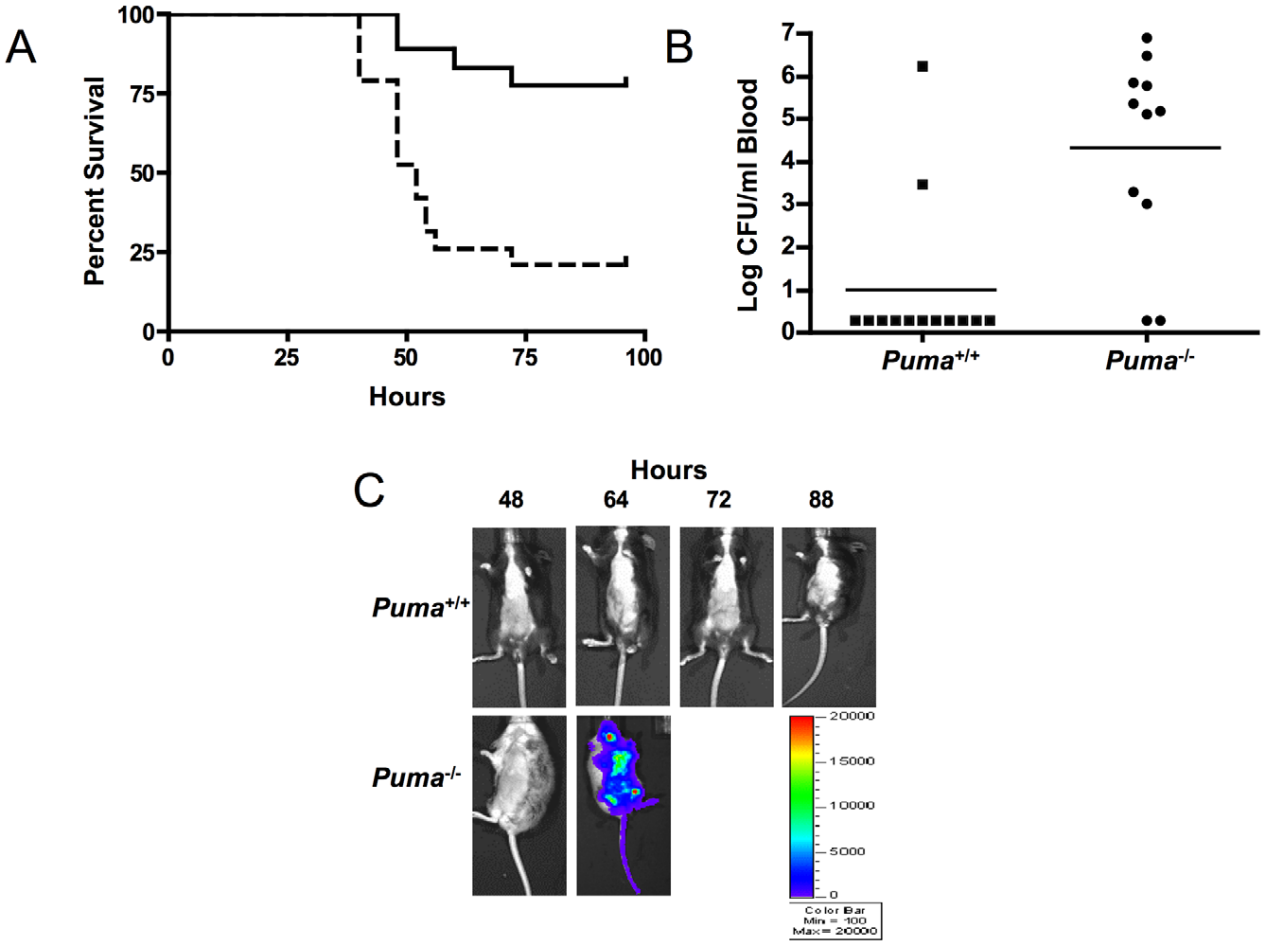
Puma-deficient mice are sensitive to pneumococcal lung infections. (A) Survival curves of *Puma* ^+/+^ (solid line; n = 19) and *Puma* ^−/−^ mice (dashed line: n = 18) after intratracheal inoculation of *S. pneumoniae* T4 (1×10^4^ cfu); p≤0.0002. Data are pooled from two experiments. (B) Mean bacterial titers in the blood of mice 24 hours after pneumococcal challenge as in A. Symbols represent individual mice; p≤0.0063. (C) Representative Xenogen images of mice challenged with luciferase-expressing *S. pneumoniae* T4X demonstrating rapid bacterial dissemination and sepsis in *Puma^−/−^* compared with *Puma* ^+/+^ animals. Color bar of luminescence intensity correlates with bacterial density.

Since *Puma*-deficient mice are protected against the lethal effects of DNA damage, we sought to determine if the same advantage was conferred in the context of an infectious challenge. *S. pneumoniae* T4 was inoculated intratracheally into *Puma* ^+/+^ and *Puma* ^−/−^ mice and the course of pneumonia and sepsis was followed over 7 days. While 80% of the *Puma* ^+/+^ mice survived the challenge, *Puma* ^−/−^ animals died rapidly with 50% succumbing by 48 hours and 84% by 96 hours (Figure 1A, p≤0.002). Mean blood titers were 1000 fold greater in *Puma* ^−/−^ mice compared to *Puma* ^+/+^ animals (Figure 1B); 84% of *Puma* ^+/+^ animals had negative blood cultures compared to only 18% of *Puma* ^−/−^ mice. Aggressive progression of infection in *Puma* ^−/−^ mice was documented further using in vivo Xenogen imaging to detect the spread of bioluminescent *S. pneumoniae* T4X. While *Puma* ^+/+^ mice rarely displayed pneumonia or sepsis, infection disseminated rapidly in *Puma* ^−/−^ mice (Figure 1C).

To determine if the survival defect in the *Puma*-deficient mice occurs during a viral infection, *Puma* ^+/+^ and *Puma* ^−/−^ mice were infected intranasally with varying infectious doses of influenza A and assayed for mortality and weight loss. There were no observable differences between the *Puma^−/−^* mice when compared to *Puma^+/+^*, suggesting that *Puma*-deficiency is not contributing an important role during influenza infection (Figure S1).

One possible basis for the failure to control bacterial infections in *Puma* ^−/−^ mice could involve poor neutrophil recruitment to the lungs following bacterial inoculation. As described previously [7], [8], circulating neutrophil and lymphocyte numbers were equivalent in *Puma* ^+/+^ and *Pum*a^−/−^ animals (Figure S2). Enumeration of leukocytes in bronchoalveolar lavage fluid indicated equivalent recruitment of macrophages and neutrophils to the lung 36 hours post infection in both genotypes. Furthermore, both intraperitoneal injection of thioglycollate or casein or direct inoculation of *S. pneumoniae* into the cerebrospinal fluid resulted in cellular influx to the same order of magnitude and with the same kinetics between *Puma* ^+/+^ and *Puma* ^−/−^ animals (data not shown). These results indicate that loss of *Puma* had no effect on phagocyte recruitment to sites of inflammation in response to a variety of stimuli.

**Figure 2:**
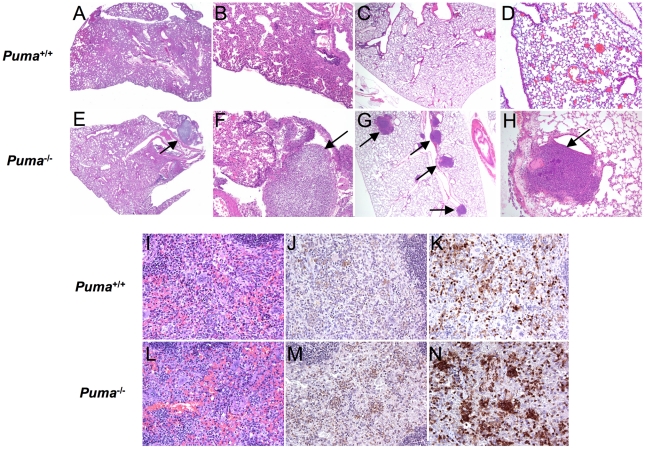
Histopathology of bacterial-infected organs. (A–H) Representative H & E stained lung sections from *Puma* ^+/+^ and *Puma^−/−^* mice at 36 hours post intranasal challenge with *S. pneumoniae* (A and E: magnification 2×; B and F: 10×) or *Staphylococcus aureus* (C and G: 4×; D and H: 10×). Arrows identify abscesses. (I–N) Spleen sections from infected mice: H&E stain (I and L); myeloperoxidase stain (J and M), and neutrophil 7/4 antigen stain (K and N) (magnification 30×). Representative sections from 4 *Puma* ^+/+^ and 3 *Puma* ^−/−^ mice.

Although neutrophils were rapidly recruited to sites of infection in *Puma* ^+/+^ and *Puma* ^−/−^ mice, histopathology indicated gross differences in the distribution of cells during pneumococcus infection. Lungs of *Puma* ^+/+^ mice demonstrated modest intra-alveolar inflammation with preservation of lung architecture (Figure 2, A and B), while *Puma* ^−/−^ mice exhibited fibrinopurulent pleuritis, extensive consolidation and loss of alveolar integrity with several mice demonstrating an accumulation of leukocytes and bacteria in prominent abscesses (Figure 2, E and F). Additionally, we evaluated lung histology during intratracheal infection with an alternate bacterial pathogen, *Staphylococcus aureus,* and determined that the infected *Puma^−/−^* mice were again more prone to abscess development than the *Puma^+/+^* infected mice (4.5 lesions/mouse vs. 2 lesions/mouse) (Figure 2, C and D vs. G and H). During pneumococcal infection, spleens from *Puma* ^−/−^ mice demonstrated greater density of neutrophils (Figure 2, L-N) when compared to those from *Puma* ^+/+^ animals (Figure 2, I-K) as confirmed by staining for myeloperoxidase and neutrophil 7/4 antigen. These findings are consistent with either decreased clearance of leukocytes from the tissues of *Puma*-null mice or a site of secondary infection.

### Increased mortality in mice deficient in the p53-Puma signaling pathway

**Figure 3:**
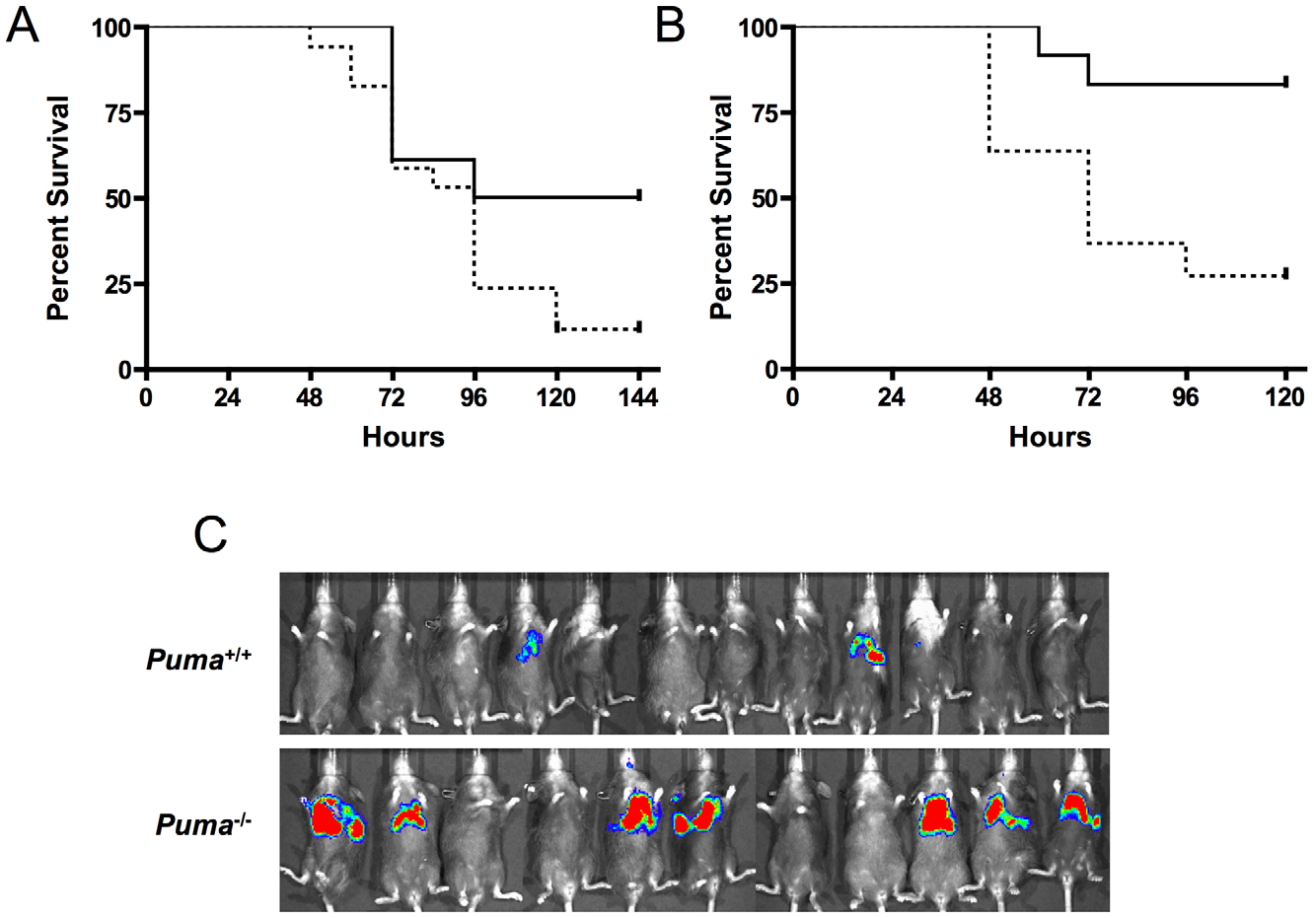
Survival of pneumococcal-infected mice deficient in p53 signaling factors. (A) Isogenic wild type (solid, n = 17) and *p53* ^−/−^ (dashed, n = 18) mice were challenged with pneumococci intranasally and survival was monitored for 7 days (p≤0.031) (mean ± SD, n = 3). (B) Isogenic wild-type (solid, n = 12) and *ATM* ^−/−^ (dashed, n = 11) mice were infected intratracheally with pneumococci and monitored for survival for 7 days (p≤0.0072). (C) Wild-type mice reconstituted with *Puma* ^+/+^ or *Puma* ^−/−^ bone marrow were analyzed by Xenogen imaging at 48 hours post-intranasal inoculation of *S. pneumoniae* T4X. Red indicates high titer bacterial pneumonia.

To further elucidate the role of Puma-induced apoptosis during pneumococcal infection, mice deficient in the upstream effector proteins ATM and p53 were challenged intranasally with pneumococci and assayed for survival. Similar to the *Puma* ^−/−^ mice, *p53^−/−^* and *ATM^−/−^* mice demonstrated increased lethality during bacterial infection (Figure 3A p≤0.031 and 3B, p≤0.0072 respectively). These findings further suggest that direct regulators of p53 activation/stabilization and its apoptotic pathway are essential for host survival during pneumococcal infection.

### Role of hematopoietic cells in aggressive sepsis in *Puma* ^−/−^ mice

The central role of hematopoietic cells in the aggressive sepsis in *Puma* ^−/−^ mice was confirmed by bone marrow transplantation. Lethally irradiated mice were transplanted with either *Puma* ^+/+^ or *Puma* ^−/−^ bone marrow. Bone marrow engraftment was confirmed at 8 weeks by flow cytometric analysis of B cells, T cells and myeloid cells and by complete blood counts to confirm that white blood cells, platelets and hemoglobin were restored to normal values (data not shown). Mice were then challenged intranasally with bioluminescent *S. pneumoniae*. Seven of eleven *Puma* ^−/−^ mice demonstrated a pronounced increase in bacterial load in the lungs 48 hours post-infection compared to two of twelve *Puma* ^+/+^ mice (Figure 3C). All *Puma* ^−/−^ mice died by 7 days while 35% of *Puma* ^+/+^ mice survived long term. These results suggest the *Puma* ^−/−^ immune defect in response to infection is intrinsic to a cell type originating from the bone marrow compartment.

### Phagocytosis and release of DNA NETs by *Puma* ^−/−^ neutrophils

**Figure 4:**
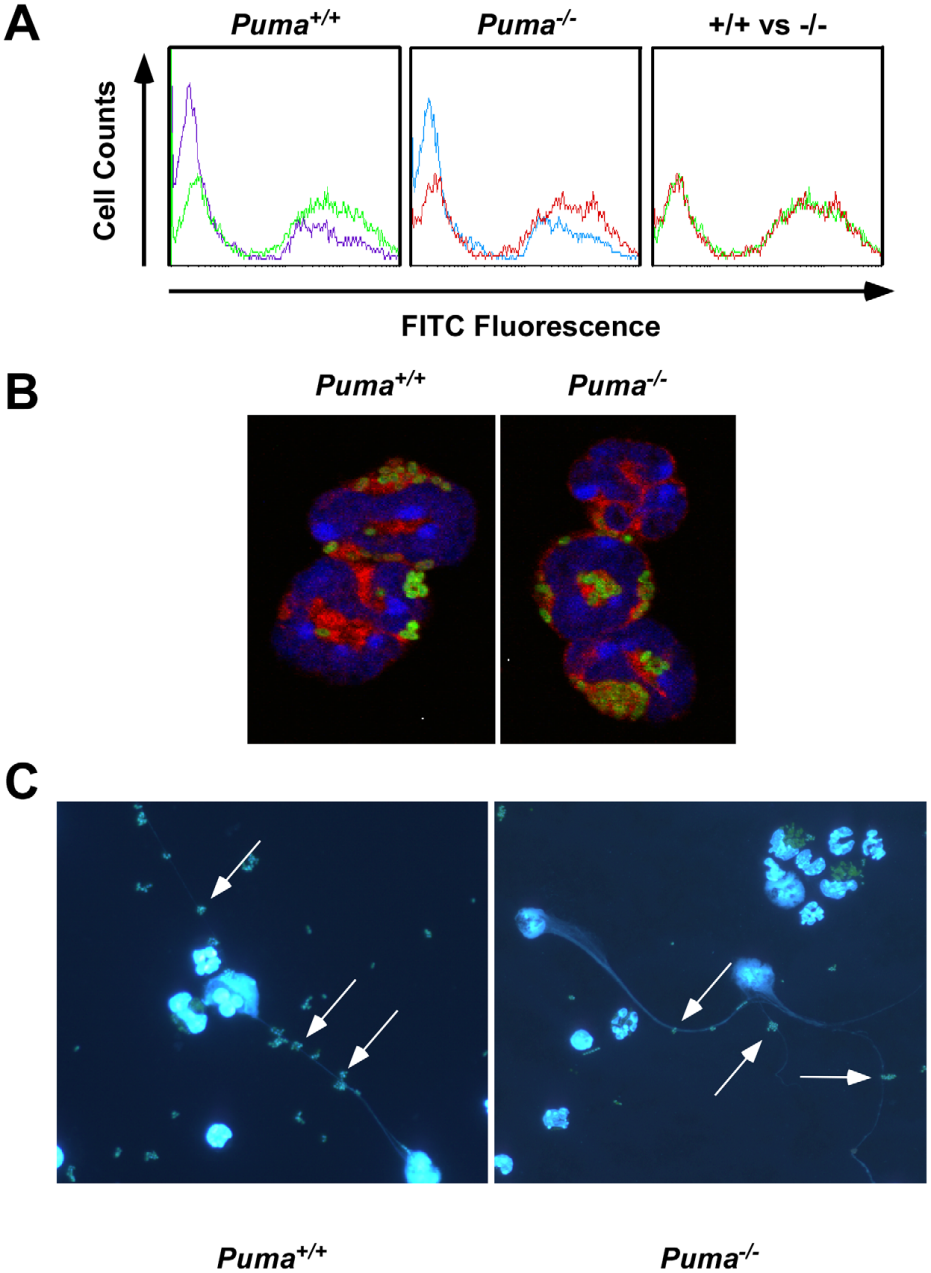
Functional analysis of neutrophil phagocytosis and NETs production. (A) Flow cytometry histograms for uptake of FITC-labeled pneumococci into peritoneal neutrophils (100∶1) over 1 hour at 37°C (green and red) or on ice (purple and blue). Shift to the right along the x axis (arrow) indicates bacterial uptake by cells. Right panel is overlay of histograms from *Puma* ^+/+^ and *Puma* ^−/−^ neutrophils incubated with bacteria at 37°C. (B) Confocal micrographs of leukocytes from panel A stained with Vybrant DiD (cytoplasm; red) and DAPI (nucleus; blue) after phagocytosis of FITC-labeled pneumococci (green). Magnification 100×. (C) Production of neutrophil extracellular traps (NETs [DNA stained blue by DAPI]: arrows indicate bacteria in NET's) by *Puma* ^+/+^ and *Puma* ^−/−^ neutrophils exposed to FITC-labeled pneumococci for 3 hours. Magnification 4×.

The failure of *Puma* ^−/−^ animals to contain bacterial pathogens, despite the presence of neutrophils in the infected tissue, could indicate a defect in bacterial phagocytosis. To test the efficacy of bacterial uptake and killing, peritoneal-recruited neutrophils and macrophages were incubated with FITC-labeled *S. pneumoniae* and bacterial/leukocyte association was quantified by fluorescence-activated cell sorting (FACS) analysis (Figure 4A and data not shown). Leukocytes from both *Puma* ^+/+^ and *Puma* ^−/−^ mice accumulated fluorescence intensity with the same kinetics and magnitude over one hour while leukocytes incubated at 4°C or with cytochalasin D to inhibit phagocytosis did not (Figure 4A, data not shown). Confocal microscopic images clearly demonstrated the presence of intracellular pneumococci in both *Puma* ^+/+^ and *Puma* ^−/−^ neutrophils (Figure 4B). Furthermore, *Puma* ^−/−^ neutrophils and macrophages phagocytosed and killed *S. pneumoniae* as efficiently as *Puma* ^+/+^ cells upon in vitro incubation with opsonized bacteria (>95.0% bacteria killed by one hour, data not shown).

In addition to phagocytosis, neutrophils release neutrophil extracellular traps (NETs) made of DNA and granules to entrap and clear extracellular bacteria [28]. To measure NET production, *Puma* ^+/+^ and *Puma* ^−/−^ peritoneal leukocytes were stimulated with either hydrogen peroxide, pneumococci, or PMA (100 nM). For both genotypes up to 70% of cells produced NETs (Figure 4C). In all, these results indicate that *Puma*-null neutrophils are functionally equivalent to *Puma* ^+/+^ with regards to migration, phagocytosis, release of NETs and bactericidal activity.

### Assessment of neutrophil apoptotic potential

**Figure 5:**
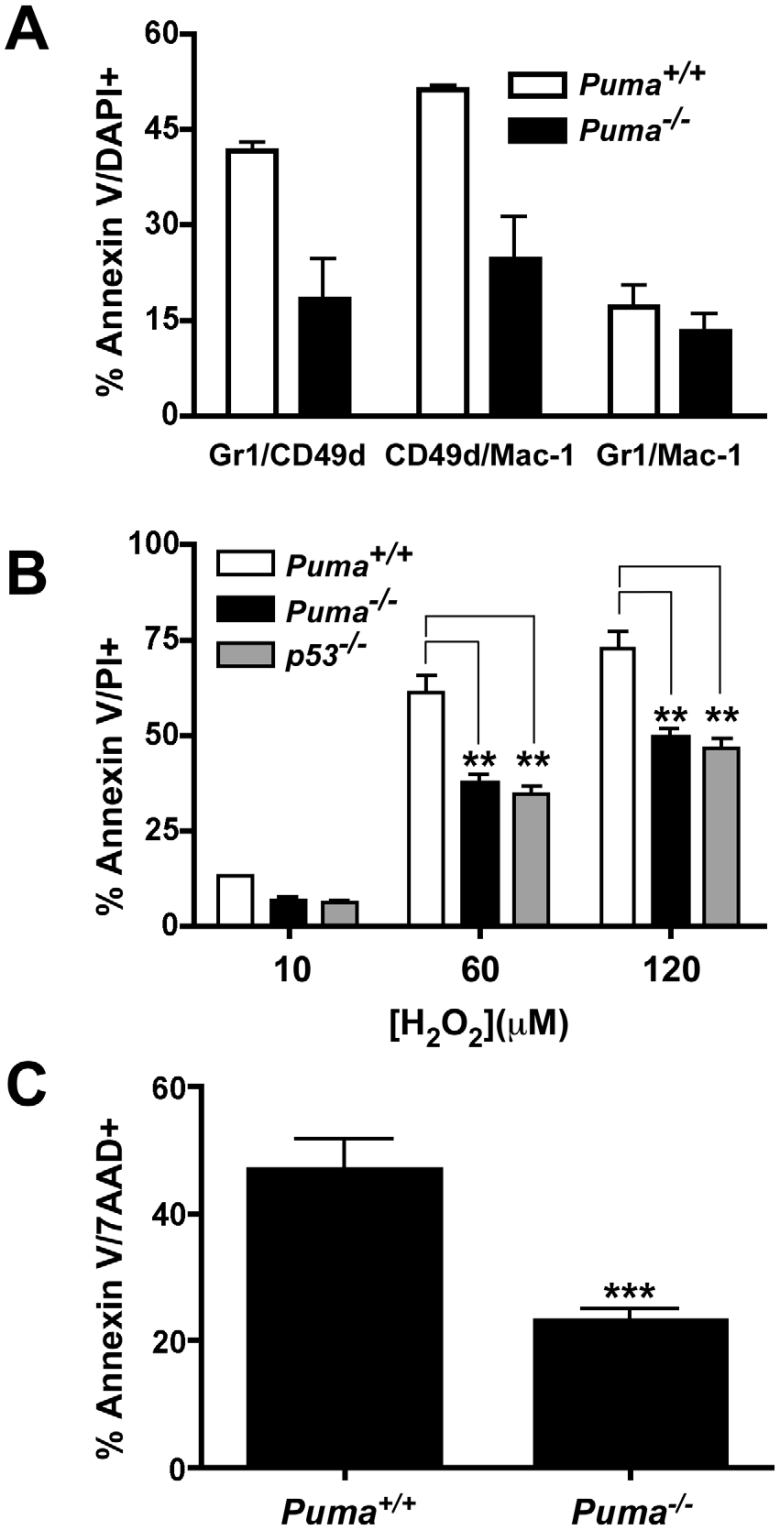
Pneumococcal-induced apoptosis of leukocyte subpopulations. (A) Percent apoptosis (AnnexinV/DAPI-positive cells) of neutrophil subpopulations from wild type (white bars) and *Puma* ^−/−^ (black bars) mice with indicated markers harvested by bronchial lavage of mice at 36 hours post-infection with *S. pneumoniae* T4 (data combines two independent experiments each with five pooled mice from both genotypes). (B) Percent apoptosis (Annexin V/propidium iodide positive) of wild type (black bars), *Puma* ^−/−^ (white bars), and *p53* ^−/−^ (grey bars) progenitor cells differentiated for 72 hours and exposed to indicated concentration of hydrogen peroxide for 3.5 hours (mean ± SD of 3 experiments). **  =  significantly different from wild type at p≤0.007 for *Puma* and <0.005 for *p53*. (C) Percent apoptosis (Annexin V/7-AAD positive) of wild type and *Puma* ^−/−^ bone marrow neutrophils treated four hours with 50 ng/ml PMA (mean ± SD of 6 experiments) ***  =  significantly different from wild type at p≤0.0009.

Neutrophils are terminally differentiated cells destined to undergo programmed cell death within 1 to 2 days after arriving at sites of infection. Apoptotic neutrophils are then cleared by macrophages to resolve inflammation [22]. This organized removal of dying neutrophils prevents the release of cytotoxic components including granules, lysosomal contents, and reactive oxygen species that can lead to further host tissue damage. The observed excess accumulation of leukocytes in lung abscesses and spleens in *Puma* ^−/−^ mice suggested a potential defect in neutrophil cell death and clearance. Given Puma's pro-apoptotic role, we hypothesized that neutrophils from *Puma* ^−/−^ mice would be defective in apoptosis, and thereby show decreased clearance from infected tissues. To test this hypothesis, *Puma* ^+/+^ and *Puma* ^−/−^ mice were challenged with *S. pneumoniae* and bronchoalveolar lavage fluid was harvested 36 hours post-infection and assessed by FACS analysis for cell type and viability in five leukocyte subsets. A panel of antibodies to cell specific markers was applied to identify neutrophils (Gr-1, Mac-1 and CD49d), macrophages (F4/80), B cells (IgM/B220), dendritic cells (CD11c/Mac-1) and T cells (CD4/CD8). Each population was assessed for apoptosis by Annexin-V/DAPI staining. The subset of neutrophils bearing CD49d^+^, which is suggested to be critical for bacterial clearance [29], showed ∼50% less apoptosis in the *Puma* ^−/−^ mice compared to wild-type mice in two independent experiments consisting of five mice for each genotype (Figure 5A). Additionally, macrophages from *Puma* ^−/−^ mice consistently showed a 10% lower rate of apoptosis compared to *Puma* ^+/+^ animals (*Puma* ^+/+^ 55±1% apoptotic cells vs. *Puma* ^−/−^ 43±2%, data not shown). These results suggest that *Puma*-deficient mice exhibit diminished apoptosis in a restricted neutrophil and macrophage population during pneumococcal infection. Consistent with the extended survival of the *Puma^−/−^* CD49d^+^ subpopulation of neutrophils, significantly more CD49d positive cells accumulated in infected lungs and abscesses were positive for CD49d cells (data not shown).

To examine the relationship between Puma and the viability and function of neutrophil subtypes in greater detail, we used two ex vivo culture systems. First, apoptotic resistance and bacterial killing were examined in primary bone marrow neutrophils. Briefly, neutrophils from *Puma* ^+/+^ and *Puma* ^−/−^ bone marrow were exposed to pneumococci in culture. Neutrophil viability was assayed by staining with Annexin V/Propidium iodide and bacterial killing was assessed by culture. *Puma* ^−/−^ neutrophils were consistently ∼30% more resistant than *Puma* ^+/+^ cells to apoptosis following incubation with bacteria (Figure S4A). Focusing specifically on the CD49d^+^ population, bacterial killing was equivalent in both *Puma* ^+/+^ and *Puma* ^−/−^ cells (Figure S4B).

Secondly, the role of Puma in neutrophil development was characterized ex vivo using immortalized wild type, *Puma* ^−/−^ or p53^−/−^ bone marrow progenitor cells. Cells were immortalized with an estrogen-regulated *hoxb8* gene and grown in stem cell factor media to promote differentiation towards the neutrophil lineage [30]. Removal of estrogen induced the hematopoietic progenitors to differentiate into mature neutrophils over the course of several days (Figure S3A). Expression of neutrophil cell surface molecules (Gr-1, Mac-1 and CD49d) was verified by flow cytometry over a 5-day time course following estrogen withdrawal. Both the timing and intensity of expression of these phenotypic markers was essentially identical between wild type and gene deficient cells (Figure S3B and data not shown). One potential mechanism where Puma could exert an anti-apoptotic effect in activated neutrophils is during oxidative stress, since Puma is a dominant regulator of apoptosis in neurons under similar conditions [31]. Oxidative stress can be triggered in neutrophils during pneumococcal infection from two primary sources: a) the *S. pneumoniae* pathogen itself is known to express significant levels of hydrogen peroxide [12], [13]; b) neutrophils produce super oxide radicals utilizing the NADPH oxidase complex resulting in increased hydrogen peroxide production, which is essential to the neutrophil's antibacterial activity [32]. To challenge the role of Puma in oxidative stress induced apoptosis, we treated the immortalized progenitor neutrophils with increasing concentrations of hydrogen peroxide. The *Puma^−/−^* and p53^−/−^ Gr1/CD49d neutrophils were significantly more resistant to this apoptotic stimulus than the wild type cells (Figure 5B). We also treated bone marrow neutrophils with phorbol 12-myristate 13- acetate (PMA, 50 ng/ml), which induces neutrophil apoptosis through respiratory burst and the production of superoxide and hydrogen peroxide [33]–[35]. Initially, we verified that *Puma^+/+^* and *Puma^−/−^* neutrophils were equivalent in the production of superoxide following PMA treatment by monitoring the oxidation of dihydrorhodamine 123 into rhodamine 123 and flow cytometry (Figure S5). Treatment of these cells with 2000 U of catalase, an enzyme that converts hydrogen peroxide into water, reduced the levels of superoxide and prevented cell death (Figure S5, and data not shown). We then treated bone marrow neutrophils from *Puma* ^+/+^ and *Puma* ^−/−^ mice with PMA, and determined by flow cytometry that loss of *Puma* significantly reduced the number of apoptotic neutrophils (∼50%) compared to wild type under differing concentrations of PMA or at varied time points (Figure 5C and data not shown). The involvement of Puma in neutrophil apoptosis was limited to oxidative stress, since treatment with Fas or staurosporine yielded equivalent cell death responses (data not shown).

## Discussion

Cells from *Puma* ^−/−^ animals and derivative primary knockout cells are remarkably resistant to apoptosis induced by a variety of insults including ionizing radiation, steroid treatment, hypoxia, and cytokine withdrawal. To be maintained in the genome, these apoptotic regulators must provide a survival advantage in response to a more frequently encountered lethal insult. Our results indicate that Puma, ATM and p53 play an essential role in the innate immune response to bacterial infections and that without any one of them, mice are extremely sensitive to these challenges.

Upon pneumococcal challenge, *Puma* ^−/−^ mice display striking lethality with 50% of challenged animals succumbing by 48 hours and average blood bacterial titers 3 logs higher than seen with wild type mice. This indicates an almost complete inability to control bacterial replication in the lungs of these mice, which is contrary to the observations made in the *Bim^−/−^* mouse (closely related BH3-only protein), since those mice appear to be more resistant to bacterial sepsis [36]. In the context of apoptosis resistance, Bim and Puma often display redundant functions during a variety of cellular insults. However, our data clearly demonstrates a drastic difference between these related BH3-only proteins. Thus, Puma and Bim appear to play opposing critical roles in the innate immune response to facilitate bacterial clearance.

Our bone marrow transplant experiments indicate that Puma's influence on outcome of infection is intrinsic to hematopoietic cells that arise from the bone marrow compartment. Of the cells originating there, neutrophils were our primary focus since they are recruited in large numbers early in response to bacterial infection and since apoptosis of neutrophils is thought to be important during the resolution of infection [22]. Functional studies with neutrophils from *Puma* ^−/−^ mice indicated that these cells are competent in normal functions such as phagocytosis, bacterial killing, and NET formation. These results argue that the uncontrolled bacterial growth and overwhelming sepsis seen in these mice are not due to non-functional neutrophils. This taken together with the formation of abscesses in the lungs and accumulation of cells in the spleen of *Puma^−/−^* mice points to an unregulated resolution of the innate response to infection. In addition, neutrophils which are unable to undergo normal apoptosis are more likely to degranulate or die of necrosis, both of which contribute to inflammation and damage to host tissues. Necrotic neutrophils and a dysregulated inflammatory response could also allow release of phagocytosed but viable bacteria and increase vascular permeability, thereby enhancing progression to sepsis. Consistent with our findings in the context of lack of expression of a pro-apoptotoic factor, Koedel et al. recently demonstrated that constitutive expression of the anti-apoptotic factor Bcl-2 led to persistent neutrophil brain infiltrates in meningitis and more severe disease [37]. These findings link the expression of an apoptosis-regulating protein with control of inflammation during pyogenic infection.

Bronchial lavages of infected *Puma* ^+/+^ and *Puma* ^−/−^ mice indicated a marked difference in apoptosis restricted to the subpopulation of neutrophils expressing CD49d and to a lesser extent, in macrophages. Other cell types were not affected, indicating that determination of cell fate in the majority of leukocytes is independent of Puma but vital to determining the lifespan of the small subset of CD49d^+^ leukocytes. This effect appears to be independent of the ability of these cells to kill bacteria. CD49d is an alpha chain integrin (VLA-4) strongly expressed early in granulocyte maturation and this expression decreases during terminal maturation [38]. Additionally, CD49d has been localized to leukocytes associated with neutrophil extravasation into the interstitial spaces of lungs during acute lung injury upon bacterial infections [39]. Tsuda et al demonstrated that the specific subpopulation of neutrophils characterized by expression of CD49d is required for resistance to *Staphylococcus aureus* infection [29]. Taken together our results corroborate the findings of Tsuda et al. suggesting a possible role for the CD49d subset of neutrophils during bacterial challenge.

Puma's involvement with DNA damage pathways may relate to its importance in protection against bacterial challenge. Activated neutrophils express large amounts of reactive oxygen species, particularly hydrogen peroxide and the hydroxyl radical. These oxidative agents are cell permeable and are known to induce cell death. Pneumococci also produce large quantities of hydrogen peroxide as a by-product of their metabolism in amounts comparable to that generated by neutrophils [40]. We demonstrated that *Puma* ^−/−^ neutrophils are more resistant to cell death when either exposed to hydrogen peroxide or when they are activated to produce hydrogen peroxide. Therefore, heightened ROS expression could be a signal that induces apoptosis in normal neutrophils while *Puma^−/−^* cells survive. ROS induced DNA-damage has been shown to trigger p53 cell death pathways and this response involved hydrogen peroxide production [41], [42]. It stands to reason that a short lived cell type that is predestined to undergo apoptosis and capable of expressing large amounts of ROS would utilize a DNA damage-induced pathway for programmed cell death. In support of this notion Marriott et al. recently reported that the NADPH oxidase deficient *gp91(phox)* ^−/−^ mice, which have limited ROS production, displayed a significant decrease in apoptosis of neutrophils recruited to lungs during bacterial pneumonia [43]. This suggests that decreased ROS production may lead to neutrophil survival, however, the diminished apoptosis does not lead to increased tissue damage and sepsis as seen in our model. It is feasible the ROS produced by the pathogen induces enough neutrophil cell death allowing for proper clearance of resident apoptotic neutrophils and a sustainable anti-inflammatory response. Therefore, only during a unique situation of pathogen challenge might the importance of such duplicity be revealed.

The importance of the DNA-damage response pathway in infection is underlined by the fact that deficiency of other proteins comprising this pathway results in a similar phenotype. We demonstrate that both *p53* ^−/−^ and *ATM* ^−/−^ mice are severely defective in controlling pneumococcal infection. These proteins both function upstream of Puma to initiate Puma-induced programmed cell death. These results bring to light a novel function for a well-characterized pathway that will significantly enhance our understanding of the innate immune response in the context of bacterial infections.

## Materials and Methods

### Bacterial strains and growth conditions

*Streptococcus pneumoniae* strains used included: serotype 4 clinical isolate TIGR4 (T4), T4R (unencapsulated mutant of TIGR4), and T4X (luciferase-expressing TIGR4). All pneumococcal strains were grown on tryptic soy agar plates supplemented with 3% sheep blood and appropriate antibiotics at 37°C in 5% CO_2_ overnight before being transferred to a defined semisynthetic casein liquid medium supplemented with 0.5% yeast extract (Sigma). Antibiotics were used at the following concentrations: chloramphenicol (5 µg/ml; Sigma) and kanamycin (400 µg/ml; Sigma). Liquid cultures were grown without aeration at 37°C in 5% CO_2_ incubator to OD_620_ of 0.4 or 0.5. *Staphylococcus aureus* NRS193 were streaked onto blood agar plates and grown overnight at 37°C. Plates were then scraped and washed with brain heart infusion media (BD Biosciences) containing 25% glycerol and frozen as concentrated stocks until used for infections.

### Animal techniques

Wild type, *p53* ^−/−^ (NM_011640), *Puma* ^−/−^ (NM_133234) and *ATM* ^−/−^ (NM_007499) mice (C57BL/6 or C57B/6/SV129 background) were anesthetized with isoflurane and challenged either intranasally (25 µl) or intratracheally (100 µl) with T4 or T4X for Xenogen IVIS 100 imaging experiments and the course of disease was followed for up to 7 days [44]. To quantify sepsis, blood samples were serially diluted in phosphate buffered saline (PBS) and plated on tryptic soy blood agar plates.

For some experiments, mice were sacrificed at 36 hours and bronchoalveolar lavage was harvested and analyzed by flow cytometry for apoptotic cells by staining with Annexin V, propidium iodide, or DAPI (Vector Laboratories). Cell types were enumerated by reactivity with a panel of antibodies specific to neutrophils (Gr-1, Mac-1 and CD49d: BD Biosciences, San Jose, CA), macrophages (F4/80: eBioscience, San Diego, CA), B cells (IgM/B220: SouthernBiotech, Birmingham, AL; BD Biosciences, San Jose, CA), dendritic cells (CD11c/Mac-1: BD Biosciences, San Jose, CA) and T cells (CD4/CD8: BD Biosciences, San Jose, CA). Apoptosis was assessed by flow cytometry using Annexin V (BD Biosciences, San Jose, CA) and DAPI (Invitrogen, Carlsbad, CA) Lungs and spleens were examined for neutrophil infiltration by immunohistochemical staining for CD49d (eBioscience, San Diego, CA), myeloperoxidase (Dako, Carpinteria, CA) and the neutrophil differentiation antigen 7/4 (Invitrogen, Carlsbad, CA). For *S. aureus* infections, bacterial stocks were diluted to 2.4×10^9^ cfu/ml and given intratracheally to mice in 100 µl volumes. Mice were followed for survival or sacrificed at 48 hours and lungs were removed for immunohistochemistry staining. Abscesses were quantified by light microscopy examination of multiple whole lung sections from both *Puma* ^+/+^ and *Puma^−/−^* mice by a pathologist blinded to the experimental design.

### Bone marrow transplantation

C67BL/6 mice (Jackson Laboratories) were lethally irradiated with 11 Gy of gamma irradiation and reconstituted with 1×10^7^ *Puma* ^+/+^ and *Puma* ^−/−^ bone marrow cells/mouse by retro-orbital injection. Mice were assayed at 8 weeks for bone marrow engraftment by complete blood counts, flow cytometry analysis of T cells (CD4+/CD8+), B cells, neutrophils and macrophages as well as RT-PCR for *Puma* deletion. Fully reconstituted mice were infected intranasally with 1×10^7^ T4X and assayed for survival and bacterial presence by Xenogen imaging.

Isolation of peritoneal macrophages, peritoneal and bone marrow neutrophils C57BL/6 mice were injected intraperitoneally with 1 ml 10% casein (Sigma) and neutrophils were isolated at 2 hours or 24 hours by peritoneal lavage using Hank's balanced salt solution (HBSS) without Ca+ or Mg+ (Invitrogen) followed by red blood cell lysis in ACK buffer (0.15 M NH_4_Cl, 10 mM KHCO_3_, 0.1 mM Na_2_EDTA; Sigma). Isolated peritoneal neutrophils were washed in Iscoves Modified Dulbecco's medium (Invitrogen) and quantified. Bone marrow neutrophils were prepared from murine femurs using discontinuous Percoll gradients (Amersham Pharmacia Biotech) as previously described [45]. Macrophages were recruited to the peritoneal cavity by IP injection of 4% thioglycollate and isolated three days post-injeciton by peritoneal lavage.

### Immortalization of neutrophil progenitors

*Puma^+/+^* and *Puma^−/−^* bone marrow cells were expanded for two days in RPMI-1640 (Invitrogen) containing 10% fetal bovine serum (FBS; Hyclone), 10 ng/ml stem cell factor (SCF; from cell culture supernatant), 10 ng/ml Interleukin 3 (Peprotech) and 20 ng/ml Interleukin 6 (Peprotech) and infected with a murine stem cell virus -based retrovirus expressing estrogen-regulated Hoxb8 as previously described [30]. Transduced non-adherent cells were serially passaged in RPMI containing 10% FBS, 1% penicillin-streptomycin, 10 ng/ml SCF and 1 µM β-estradiol (Sigma) to expand for immortalized myeloid progenitors for 20 days, by which time control marrow cultures had stopped dividing. Progenitors were induced to undergo differentiation into neutrophils upon β-estradiol removal and culturing in the presence of SCF. Bacterial phagocytosis/lysis assays Murine neutrophils were isolated by peritoneal lavage 24 hours after injection of 10% casein (Sigma). After red cell lysis (see above), neutrophils were resuspended in HBSS with Ca^2+^ and Mg^2+^ modified with 0.1% gelatin (Sigma) and 10% fresh mouse serum, followed by a 1 hour rotated incubation at 37°C or 0°C with various ratios of FITC-labeled-bacteria:neutrophils as described in the text. Phagocytosis was assessed by flow cytometry quantitating FITC+ cells. Results were confirmed by visualizing FITC labeled bacteria in cells stained with Vybrant DiD (Invitrogen) and DAPI using fluorescence microscopy. Bacterial was determined by washing the neutrophils in PBS, lysing the cells in sterile H_2_O and determining bacterial growth from serial dilutions of lysates grown on blood agar plates [46].

### Ex vivo neutrophil characterization

Purified bone marrow neutrophils from *Puma* ^+/+^ or *Puma* ^−/−^ mice were suspended in HBSS with Ca^2+^ and Mg^2+^ modified with 0.1% gelatin and incubated for 1 hour with serum opsonized *S. pneumoniae* T4R at bacteria/neutrophil ratios of 1∶10, 1∶1, or 5∶1. Cells were then washed in PBS with 1% FBS, resuspended in RPMI-1640 supplemented with 10% FBS and incubated at 37°C in 5% CO_2_ for 3 hours. 3×10^6^ neutrophils were washed and stained for 15 min with Annexin V-FITC (BD Biosciences). Propidium iodide (BD Biosciences) was added and cells were analyzed by FACS for specific apoptosis. Specific apoptosis was calculated by the formula: (test – control)/(100 – control) ×100. The test sample was incubated with bacteria while the control received no bacteria but was otherwise treated identically.

### NET characterization

Peritoneal neutrophils were recruited by intraperitoneal injection of 10% casein (Sigma) and harvested by peritoneal lavage. The cells were washed and resuspended in PBS with Ca^2+^ and Mg^2+^ (Invitrogen), and allowed to adhere for 20 min. to poly-L-lysine coated coverslips (Sigma-Aldrich) in 6-well plates. Neutrophils were then treated with either serum-opsonized heat killed *S. pneumoniae* T4R (100 bacteria/neutrophil; labeled with FITC) or H_2_O_2_ (125 µM; Sigma) for 3 hours or PMA (100 nM) for 16 hours in RPMI media with 10%FBS. After stimulation, cover slips were fixed with 3% paraformaldehyde and mounted onto slides with DAPI-Vectormount (Vector Labs) or PL2-3 anti-histone antibody (courtesy of Dr. A Zychlinsky). DNA stained NETs were visualized with a fluorescence microscope.

### H_2_O_2_ stress-induced apoptosis

Immortalized neutrophil progenitors were washed free of estrogen and allowed to differentiate for 3 days. Cells were then washed with fresh RPMI and suspended at 5×10^5^ cells/ml in 2 ml RPMI (10% FBS) containing hydrogen peroxide (Sigma) at 0, 10, 60, or 120 µM for 3.5 hours. Cells were then washed once with PBS (1% FBS), stained with Annexin V and propidium iodide and assayed by flow cytometry for apoptosis.

Bone marrow neutrophils were isolated (described above) and treated in vitro for 30 minutes or four hours with 50 ng/ml phorbol 12-myristate 13-acetate (Sigma-Aldrich) in RPMI 1640 medium supplemented with 10% fetal bovin serum (Hyclone), 2 mM L-glutamine and penicillin (100 IU/ml) and streptomycin (100 ug/ml) at 37°C in 5% CO_2_. Cells were assayed by flow cytometry for superoxide production using Dihydrorhodamine 123 (Calbiochem) or for apoptosis by Annexin V/7-Amino Actinomycin D (BD Biosciences). StatisticsKaplan-Meir survival curves were tested for significant difference utilizing the logrank test. Additional statistical analyses were performed by the Student's *t*-test. In all cases, p<0.05 was considered as statistical significance.

### Ethics statement

All experiments involving animals were performed with prior approval of and in accordance with guidelines of the St. Jude Institutional Animal Care and Use Committee. The St Jude laboratory animal facilities have been fully accredited by the American Association for Accreditation of Laboratory Animal Care. Laboratory animals are maintained in accordance with the applicable portions of the Animal Welfare Act and the guidelines prescribed in the DHHS publication, Guide for the Care and Use of Laboratory Animals.

## Supporting Information

Figure S1 Influenza infection of *Puma* ^+/+^ and *Puma* ^−/−^ mice. Mice were infected intranasally with the indicated doses of influenza strain X31 and followed for survival and weight loss over time (*Puma* ^+/+^: (WT) solid line; *Puma* ^−/−^: (KO) dashed line). Each line is an individual mouse.
(0.12 MB TIF)

Figure S2 Quantification of neutrophils and macrophages from *Puma* ^+/+^ and *Puma* ^−/−^ mice. Neutrophils (Gr1+/Mac1+) and macrophages (F480+/Mac1+) were quantified by flow cytometry from bone marrow and spleen of *Puma* ^+/+^ and *Puma* ^−/−^ mice (n = 5; p =  not significant).
(0.13 MB TIF)

Figure S3 Differentiation of *Puma* ^−/−^ progenitor cells in vitro. (A) Wright-Giemsa stained cytospins of *Puma* ^+/+^ and *Puma* ^−/−^ neutrophil progenitors undifferentiated (+ estrogen) or differentiated (- estrogen) for 3 and 6 days. Multi-lobed nucleus characteristic of neutrophils is evident upon differentiation. (B) Flow cytometric histograms of *Puma* ^+/+^ and *Puma^−/−^* neutrophil progenitors during differentiation demonstrating expression of cell surface markers Gr-1 and CD49d over time.
(2.08 MB TIF)

Figure S4 Apoptosis and function of neutrophils incubated with *S. pneumoniae*. (A) Bone marrow neutrophils (n) were incubated in different ratios with T4R pneumococcus (b) for 1 hr; 4 hrs later cells were stained with AnnexinV/Propidium Iodide and quantified for apoptosis by flow cytometry (mean ± SD for 4 experiments). *Puma* ^+/+^: white bars; *Puma* ^−/−^: black bars. Data are presented as the mean ± SEM of four independent experiments: **p≤0.008. (B) CD49d+ bone marrow neutrophils unprimed (TNF-) or primed (TNF+) were incubated with bacteria for 1 hour at 37°C and the percentage of bacterial killing was quantitated by culture.
(0.06 MB TIF)

Figure S5 Analysis of superoxide production in *Puma* ^+/+^ and *Puma* ^−/−^ bone marrow neutrophils. Bone marrow neutrophils from *Puma^+/+^* and *Puma^−/−^* were treated for 30 minutes with 50 ng/ml of PMA, either with or without 2000 U/ml of catalase, then assayed for the presence of superoxide using dihydrorhodamine 123 and flow cytometry (mean ± SD of 6 experiments).
(0.04 MB TIF)
