# Supplementary figures and images for: The p53-Target Gene Puma Drives Neutrophil-Mediated Protection against Lethal Bacterial Sepsis
Source: PLoS Pathog. 2010 Dec 23;6(12):e1001240. doi: 10.1371/journal.ppat.1001240 (PMC3009602; doi:10.1371/journal.ppat.1001240)

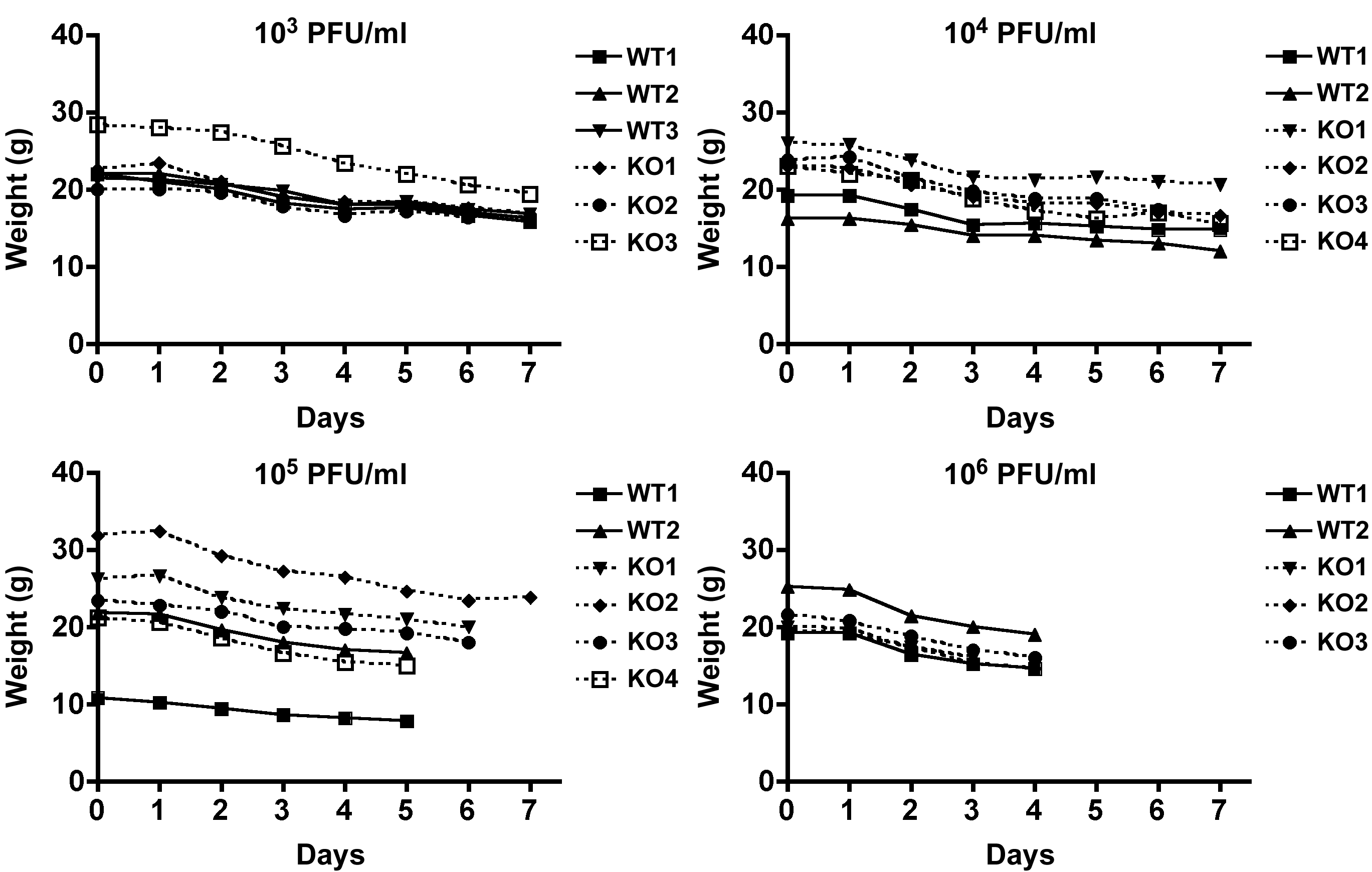

Supplement: Figure S1 — Influenza infection of Puma +/+ and Puma −/− mice. Mice were infected intranasally with the indicated doses of influenza strain X31 and followed for survival and weight loss over time (Puma +/+: (WT) solid line; Puma −/−: (KO) dashed line). Each line is an individual mouse. (0.12 MB TIF) [file ppat.1001240.s001.tif]

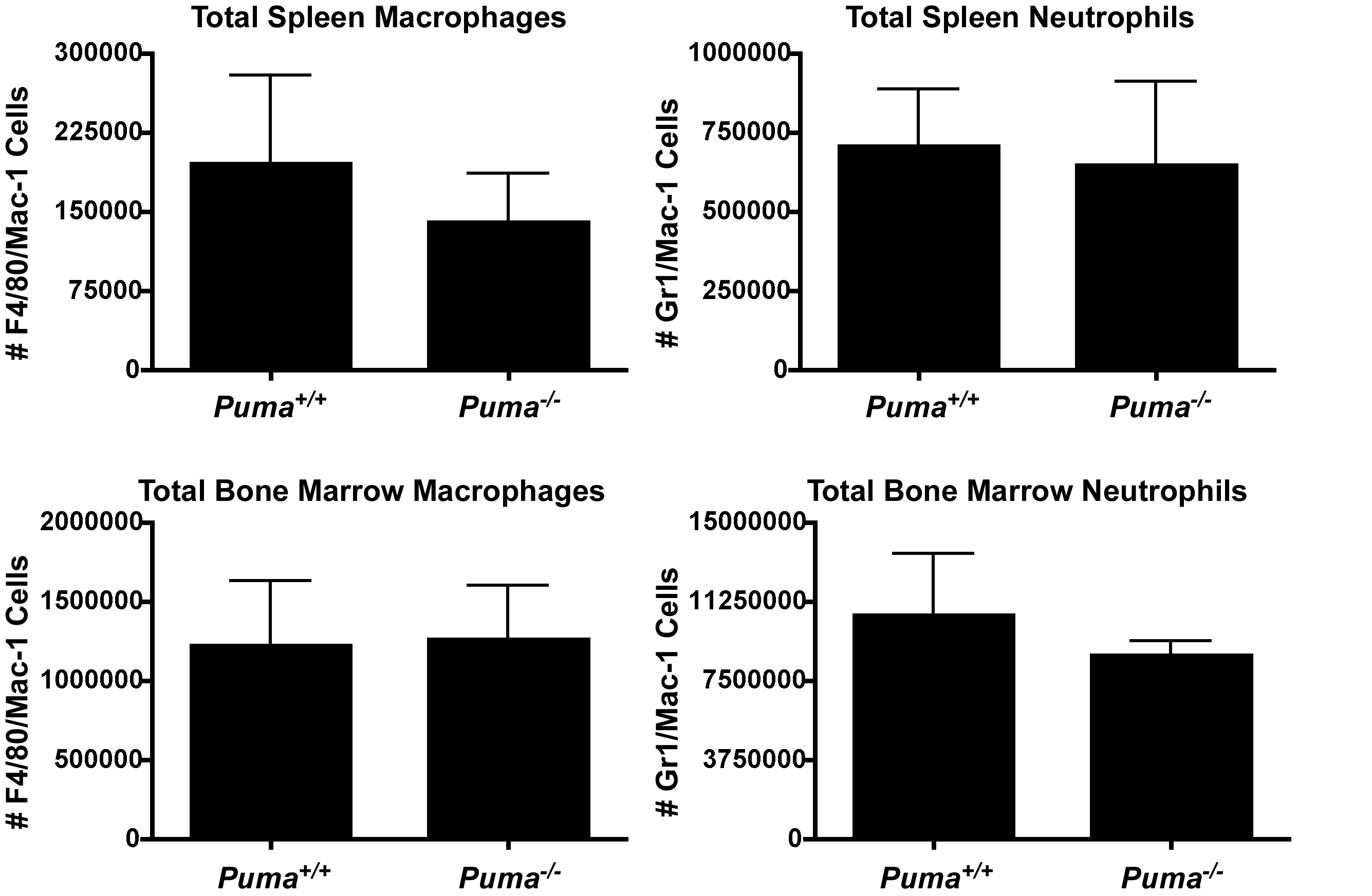

Supplement: Figure S2 — Quantification of neutrophils and macrophages from Puma +/+ and Puma −/− mice. Neutrophils (Gr1+/Mac1+) and macrophages (F480+/Mac1+) were quantified by flow cytometry from bone marrow and spleen of Puma +/+ and Puma −/− mice (n = 5; p = not significant). (0.13 MB TIF) [file ppat.1001240.s002.tif]

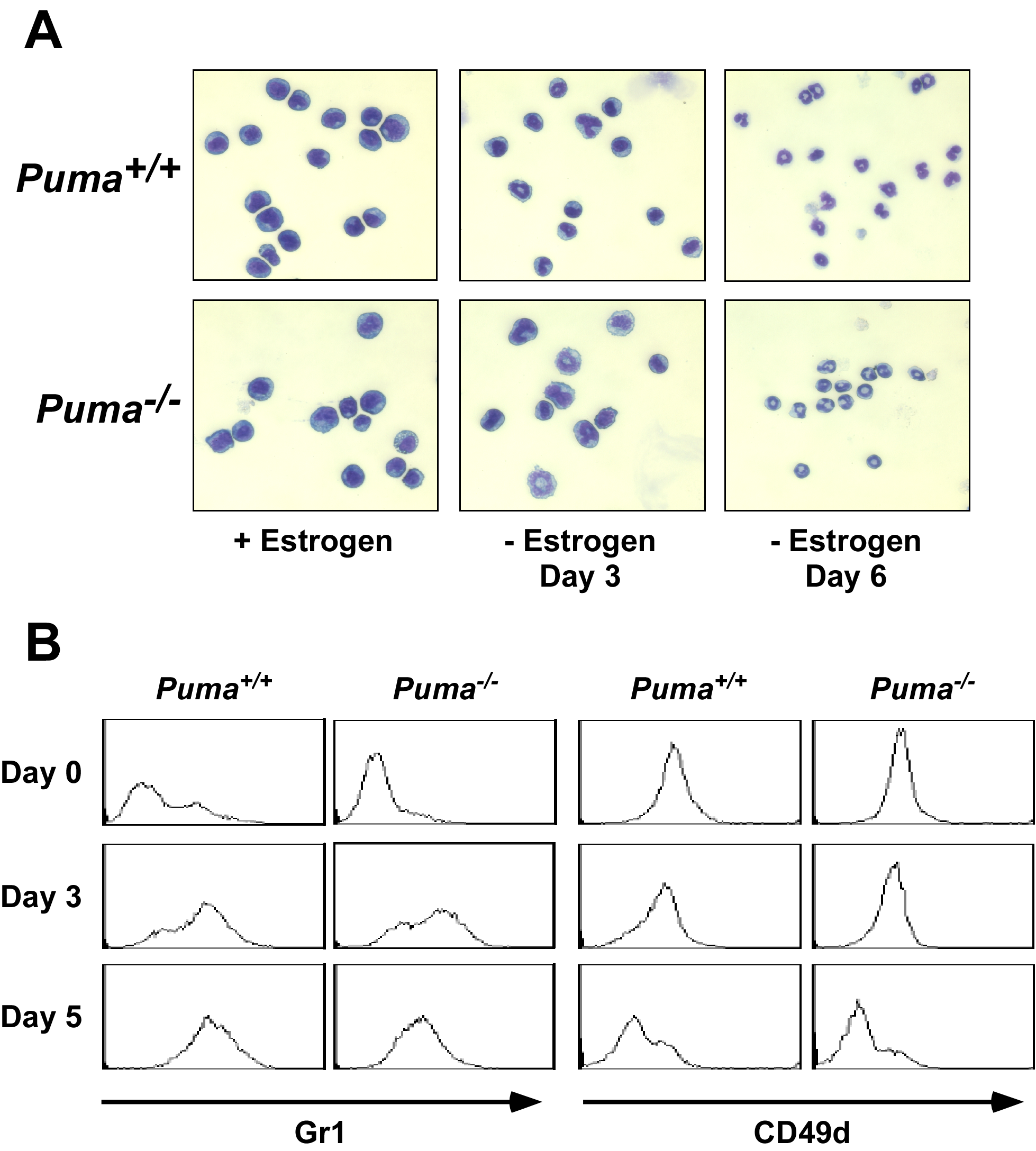

Supplement: Figure S3 — Differentiation of Puma −/− progenitor cells in vitro. (A) Wright-Giemsa stained cytospins of Puma +/+ and Puma −/− neutrophil progenitors undifferentiated (+ estrogen) or differentiated (- estrogen) for 3 and 6 days. Multi-lobed nucleus characteristic of neutrophils is evident upon differentiation. (B) Flow cytometric histograms of Puma +/+ and Puma−/− neutrophil progenitors during differentiation demonstrating expression of cell surface markers Gr-1 and CD49d over time. (2.08 MB TIF) [file ppat.1001240.s003.tif]

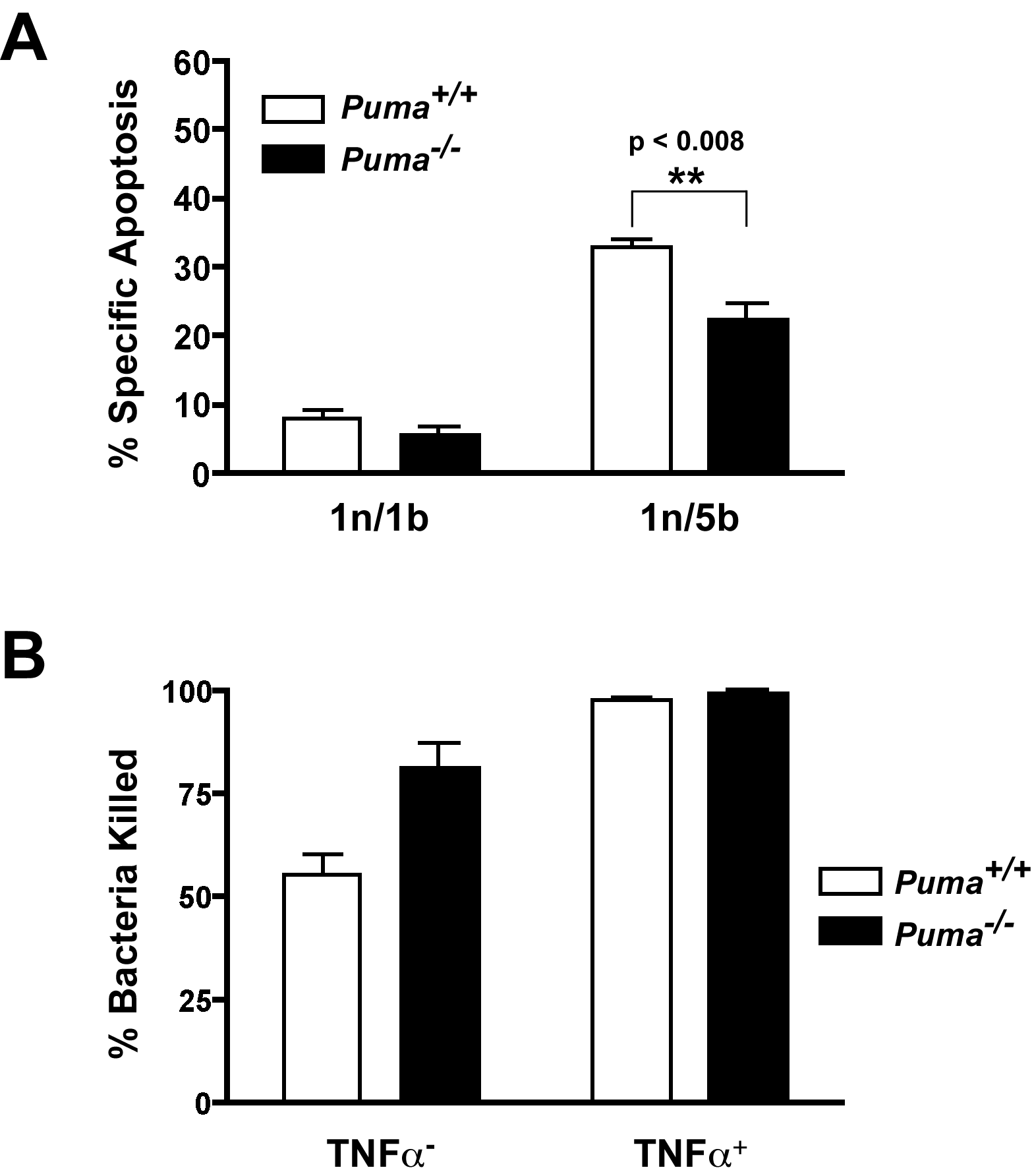

Supplement: Figure S4 — Apoptosis and function of neutrophils incubated with S. pneumoniae. (A) Bone marrow neutrophils (n) were incubated in different ratios with T4R pneumococcus (b) for 1 hr; 4 hrs later cells were stained with AnnexinV/Propidium Iodide and quantified for apoptosis by flow cytometry (mean ± SD for 4 experiments). Puma +/+: white bars; Puma −/−: black bars. Data are presented as the mean ± SEM of four independent experiments: **p≤0.008. (B) CD49d+ bone marrow neutrophils unprimed (TNF-) or primed (TNF+) were incubated with bacteria for 1 hour at 37°C and the percentage of bacterial killing was quantitated by culture. (0.06 MB TIF) [file ppat.1001240.s004.tif]

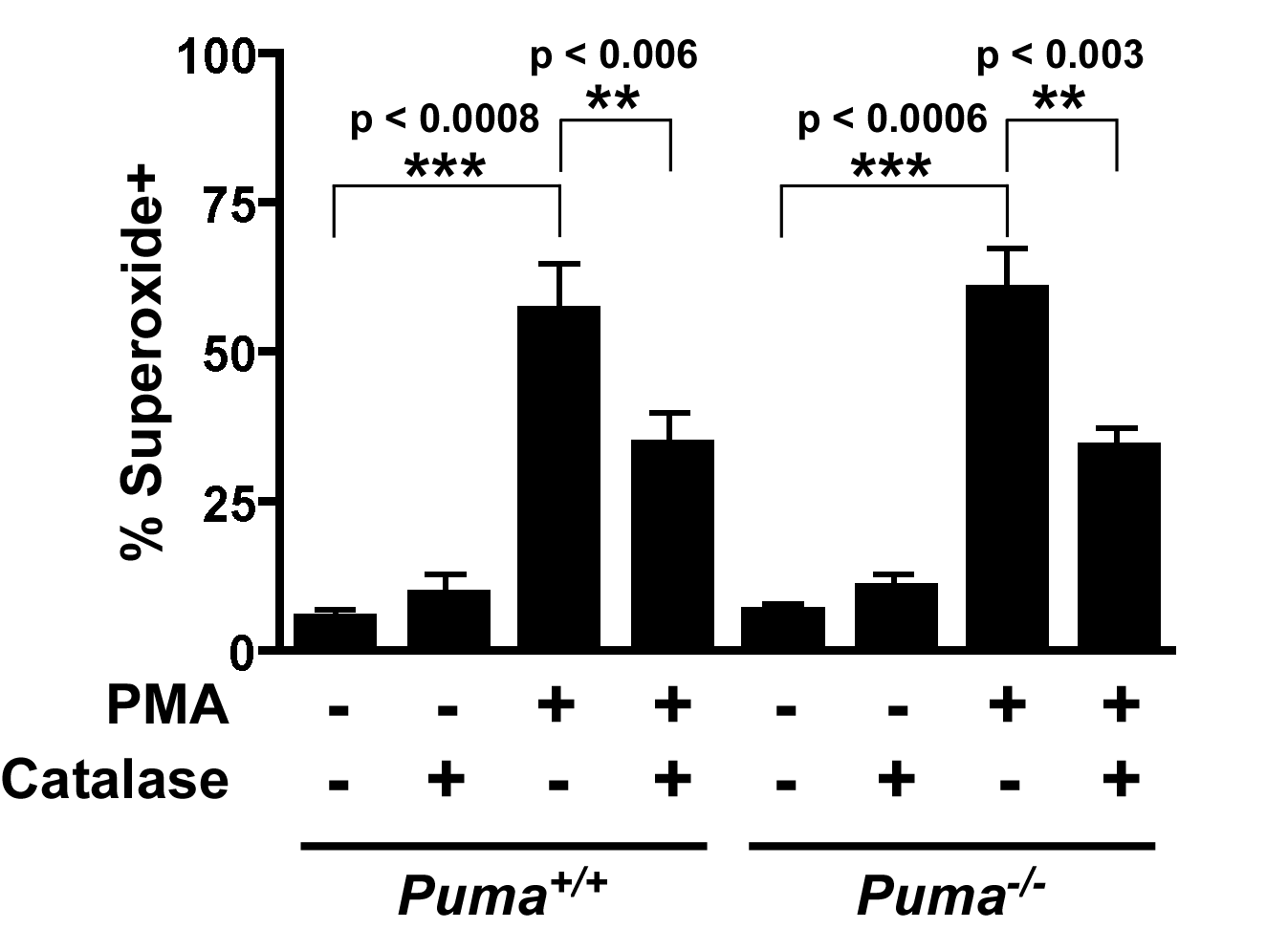

Supplement: Figure S5 — Analysis of superoxide production in Puma +/+ and Puma −/− bone marrow neutrophils. Bone marrow neutrophils from Puma+/+ and Puma−/− were treated for 30 minutes with 50 ng/ml of PMA, either with or without 2000 U/ml of catalase, then assayed for the presence of superoxide using dihydrorhodamine 123 and flow cytometry (mean ± SD of 6 experiments). (0.04 MB TIF) [file ppat.1001240.s005.tif]
